# Fabrication of Nanocrystals for Enhanced Distribution of a Fatty Acid Synthase Inhibitor (Orlistat) as a Promising Method to Relieve Solid Ehrlich Carcinoma-Induced Hepatic Damage in Mice

**DOI:** 10.3390/ph17010096

**Published:** 2024-01-10

**Authors:** Jawaher Abdullah Alamoudi, Thanaa A. El-Masry, Mohamed Nasr, Ismail T. Ibrahim, Hanaa A. Ibrahim, Hebatallah M. Saad, Maysa M. F. El-Nagar, Samar Zuhair Alshawwa, Amal Alrashidi, Enas I. El Zahaby

**Affiliations:** 1 Department of Pharmaceutical Sciences, College of Pharmacy, Princess Nourah bint Abdulrahman University, P.O. Box 84428, Riyadh 11671, Saudi Arabia; jaalamoudi@pnu.edu.sa (J.A.A.); szalshawwa@pnu.edu.sa (S.Z.A.); aaalrashidi@pnu.edu.sa (A.A.); 2 Department of Pharmacology and Toxicology, Faculty of Pharmacy, Tanta University, Tanta 31527, Egypt; thanaa.elmasri@pharm.tanta.edu.eg (T.A.E.-M.); hanaa.abdelkareem@pharm.tanta.edu.eg (H.A.I.); 3 Department of Pharmaceutics, Faculty of Pharmacy, Delta University for Science and Technology, Gamasa 35712, Egypt; m2naser@yahoo.com (M.N.); enas.elzahabi@deltauniv.edu.eg (E.I.E.Z.); 4 Department of Pharmaceutics and Industrial Pharmacy, Faculty of Pharmacy, Helwan University, Cairo 11790, Egypt; 5 Labeled Compounds Department, Hot Laboratory Centre, Egyptian Atomic Energy Authority, Cairo 13759, Egypt; ismailtaha2018@gmail.com; 6 Department of Pharmacy, Al-Huda University College, Anbar 31001, Iraq; 7 Department of Pathology, Faculty of Veterinary Medicine, Matrouh University, Cairo 51511, Egypt; heba.magdy@mau.edu.eg

**Keywords:** bioavailability, biodistribution, nanocrystals, liver damage, orlistat, radiolabeling, solid Ehrlich carcinoma

## Abstract

Background: Orlistat (ORL) is an effective irreversible inhibitor of the lipase enzyme, and it possesses anticancer effects and limited aqueous solubility. This study was designed to improve the aqueous solubility, oral absorption, and tissue distribution of ORL via the formulation of nanocrystals (NCs). Methods: ORL-NC was prepared using the liquid antisolvent precipitation method (bottom-up technology), and it demonstrated significantly improved solubility compared with that of the blank crystals (ORL-BCs) and untreated ORL powder. The biodistribution and relative bioavailability of ORL-NC were investigated via the radiolabeling technique using Technetium-99m (^99m^Tc). Female Swiss albino mice were used to examine the antitumor activity of ORL-NC against solid Ehrlich carcinoma (SEC)-induced hepatic damage in mice. Results: The prepared NCs improved ORL’s solubility, bioavailability, and tissue distribution, with evidence of 258.70% relative bioavailability. In the in vivo study, the ORL-NC treatment caused a reduction in all tested liver functions (total and direct bilirubin, AST, ALT, and ALP) and improved modifications in liver sections that were marked using hematoxylin and eosin staining (H&E) and immunohistochemical staining (Ki-67 and ER-α) compared with untreated SEC mice. Conclusions: The developed ORL-NC could be considered a promising formulation approach to enhance the oral absorption tissue distribution of ORL and suppress the liver damage caused by SEC.

## 1. Introduction

The lipase inhibitor orlistat (ORL) prevents the fatty acid synthase (FAS) enzyme from activating its thioesterase domain, which is especially upregulated in many tumor cells, and its inhibition has been shown to suppress tumor growth and metastasis [1,2]. Several studies have revealed elevated FAS in different cancer types compared with normal tissues [3,4]. This metabolic mechanism gives cells more energy, which encourages the development of malignant phenotypes [5].

The anticancer effect of ORL is dose-dependent [1,6]. Recent studies revealed the effective role of ORL as an anticancer agent, exerting anticancer effects through different mechanisms, such as the induction of tumor cell apoptosis, the inhibition of tumor angiogenesis, the interference with the tumor cell cycle, and the induction of tumor cell ferroptosis [7,8]. Moreover, many other studies have demonstrated the potential for orlistat to work in concert with traditional chemotherapy drugs to increase their efficacy and minimize side effects in various cancer models [9,10].

Because of its waxy form and extreme hydrophobicity (predicted log P is 8.1), low melting point (40–50 °C), and poor chemical stability, ORL is regarded as an impractical and difficult-to-manage substance from a technological standpoint.

According to BCS, ORL is classified as a class II drug, with its limited solubility throughout physiological pH ranges and dissolution rate explaining its low therapeutic effectiveness, regardless of whether it is administered locally or systematically. As a result, higher dosages must be given, which might elevate the risk of major adverse effects [11]. Therefore, novel approaches should be used to develop a new formulation of ORL acting as a FASN inhibitor for cancer therapy. Several types of research methods have been performed to enhance the solubility of ORL, including extrusion, spheronization, and micronization. The enhanced solubility of ORL represents a new potential for using ORL in combination with conventional chemotherapy in obesity management [11,12].

Two techniques, “bottom-up technology” and “top-down technology,” are used to prepare nanosuspensions. Bottom-up technology is a method of assembling nanoparticles (NPs), such as through precipitation, microemulsion, and melt emulsification. Meanwhile, top-down technology entails the disintegration of larger particles into smaller ones, such as through high-pressure homogenization and milling [13]. One of the most successful pharmaceutical nanotechnology approaches is drug nanosuspensions (nanocrystals (NCs)) [14]. NCs are finely dispersed drug particles suspended in an aqueous solution ranging from 1 to 1000 nm in size. Moreover, considering the oral route, NCs are permeated with fast dissolution and bioadhesive properties [11]. The only component of a nanosuspension is the medicine itself; an NC addresses low solubility and bioavailability issues and modifies the medication pharmacokinetics, enhancing medicinal efficacy and safety [12]. Poorly soluble medications can now be administered intravenously without blocking blood capillaries thanks to NCs, which have emerged as a low-cost delivery system for drugs that are poorly soluble in water [15]. Stabilizers may enhance NC stability and efficient particle size reduction in addition to in vivo and in vitro performance [16].

Technetium-99m (^99m^Tc) is a relatively inexpensive radioisotope that is most frequently used in SPECT (single-photon emission computerized tomography) diagnostic nuclear medicine. Its popularity is due to it having a short half-life (6 h), being easily obtained from Mo-99generators and having a decay energy of 140.5 keV, which falls within the range of a gamma camera. SPECT using Technetium-99m is widely employed for imaging of the brain, bones, lungs, kidneys, thyroid, heart, gall bladder, liver, spleen, bone marrow, salivary and lachrymal glands, blood pool, and cancer [17]. This radioisotope can generate pure gamma rays with an energy of 140.5 keV, does not release charged-particle radiation, can be found as a free carrier, and may attach to a wide variety of substances [18].

Ehrlich tumors are considered spontaneous murine mammary adenocarcinomas [19]. They are a transplantable tumor model that has been used to easily investigate the anticancer properties of several chemical substances; however, they can also cause oxidative stress and liver damage [20]. One of the most crucial bodily organs, the liver, is the master regulator of several biochemical and physiological processes. Numerous studies have shown that tumor angiogenesis is associated with hepatic fibrosis and inflammation in chronic liver diseases and has features that contribute to hepatocellular carcinoma development [21,22]. Angiogenesis is an extremely complicated physiological process carefully controlled by various growth hormones, inflammatory cytokines, and physiological hypoxia [23]. Tumor angiogenesis, the growth of new blood vessels to nourish the rapid proliferation of tumor cells, is a serious stage in the tumor metastatic process of nearly all malignancies [24]. A detailed investigation of the correlation between cytokines and tumor angiogenesis is also crucial for developing effective treatment for liver fibrosis and inflammation [25].

Radiation and chemotherapy are the most common cancer treatments to trigger apoptosis in cancer cells [26]. Tamoxifen is a potent anticancer drug that is known to interfere with the increased estrogen activity of malignant mammary gland cells. It achieves its antiestrogenic action by interacting with the estrogen receptors on breast tissue and blocking the binding of estrogen molecules to these receptors [27]. It is used in this study as conventional chemotherapy and for comparison with the anticancer effect of ORL and its nanoformulation [9].

Therefore, the aim of this study is to fabricate an economical and simple formulation of one of the most challenging hydrophobic waxy drugs—ORL. This study investigates the tissue distribution of ORL in different body organs using the radiolabeling technique and analyzes its anticancer effect on mice to support preclinical studies on the beneficial effects of ORL as an effective anticancer agent.

## 2. Results

### 2.1. Saturated Aqueous Solubility

The results of saturated aqueous solubility of ORL-NC and ORL-BC showed that the solubility was 363.5 ± 43.37 µg/mL and 60.2 ± 11.24 µg/mL for ORL-NC and ORL-BC, respectively. Repeating the same procedures in 0.1 N HCl, the nanocrystals displayed higher solubility than the control crystals; the saturated aqueous solubility in 0.1 N HCl was 457.57 ± 90.35 μg/mL and 36.77 ± 5.29 μg/mL for ORL-NC and ORL-BC, respectively. The results of ORL saturated solubility in water and 0.1 N HCL were below the limit of quantitation of the applied analytical method (ORL is practically insoluble (0.49 ± 0.12 µg/mL)) [6] (Table 1).

### 2.2. Particle Size and Zeta Potential Analysis

**Figure 1:**
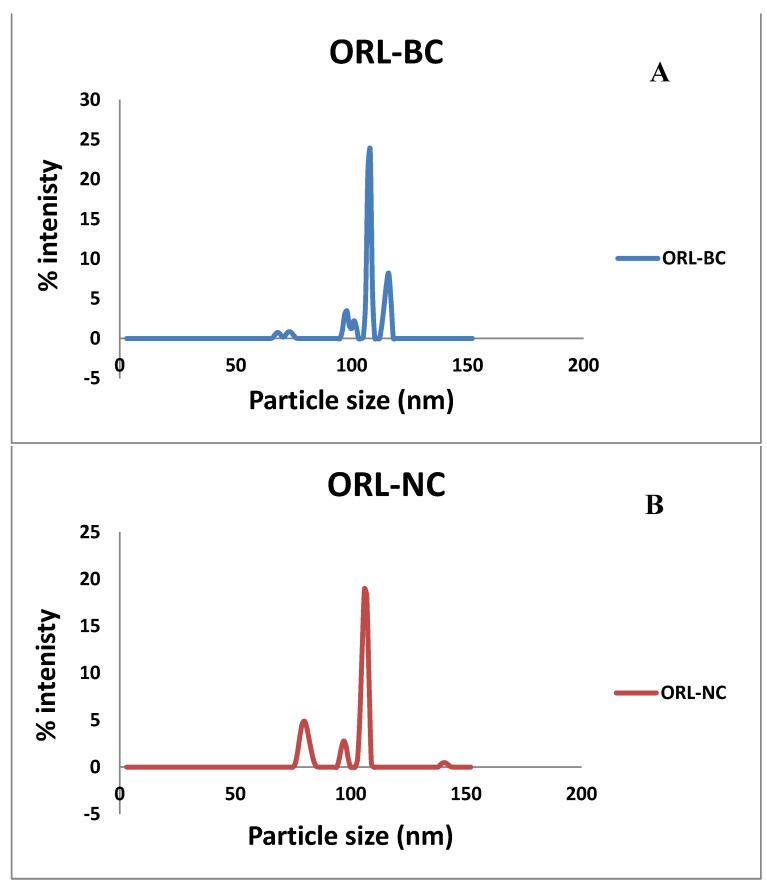
Particle size distribution.

As illustrated in Table 1, the average particle size was 329.59 ± 120.75 nm and 557 ± 189.63 nm for ORL-NC and ORL-BC, respectively (Table 1). The zeta potential was quite similar, with a negative value of −27.04 ± 5.5 mV and −26.19 mV for ORL-NC and ORL-BC, respectively (Table 1). The polydispersity index was less than 0.5 (Table 1 and Figure 1).

### 2.3. Scanning Electron Microscope (SEM)

**Figure 2:**
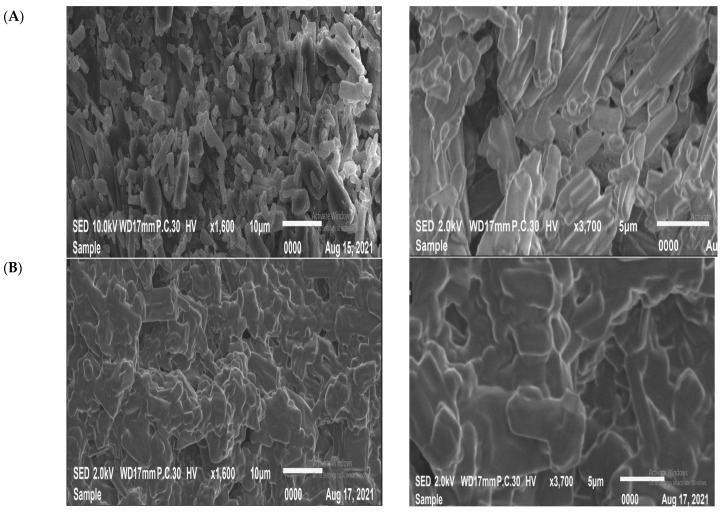
Scanning electron microscopy images (SEM) of ORL-NC (**A**) and ORL (**B**).

The morphologies and sizes of various ORL-NC are clearly different, as indicated by the SEM pictures of morphology and particle size in Figure 2. ORL appears to be primarily composed of non-homogenous rod-like irregular shapes. The ORL-NC appears as uniform rode-aggregated particles that are composed of small, well-defined rods.

### 2.4. Differential Scanning Calorimetry (DSC)

**Figure 3:**
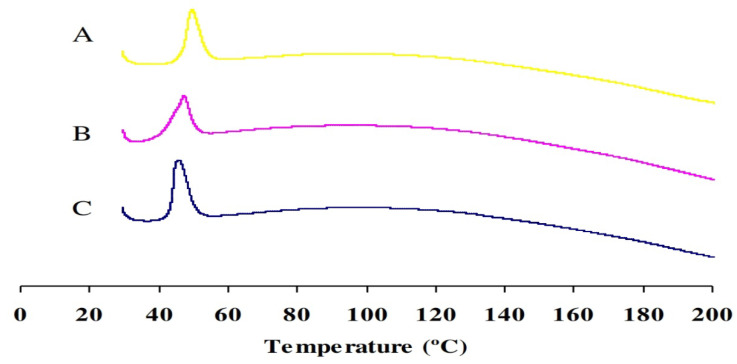
DSC thermograms of ORL (**A**), ORL-NC (**B**), and ORL-BC (**C**).

DSC thermograms of ORL, ORL-NC, and ORL-BC were investigated; all thermograms showed endothermic peaks representing the melting point (T_m_) but with different intensities. The most prominent intensity was found in the ORL thermogram at 45.67 °C and the least prominent intensity was found in the ORL-NC thermogram with a tiny shift to higher T_m_ at 47.22 °C (Table 2 and Figure 3).

### 2.5. X-ray Diffraction

**Figure 4:**
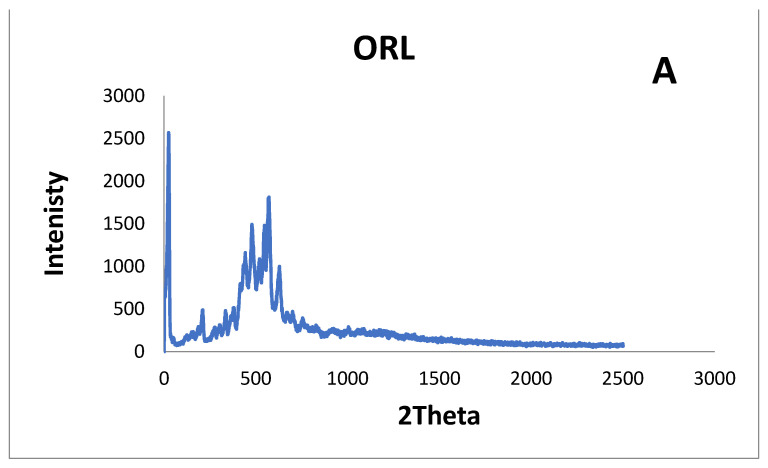

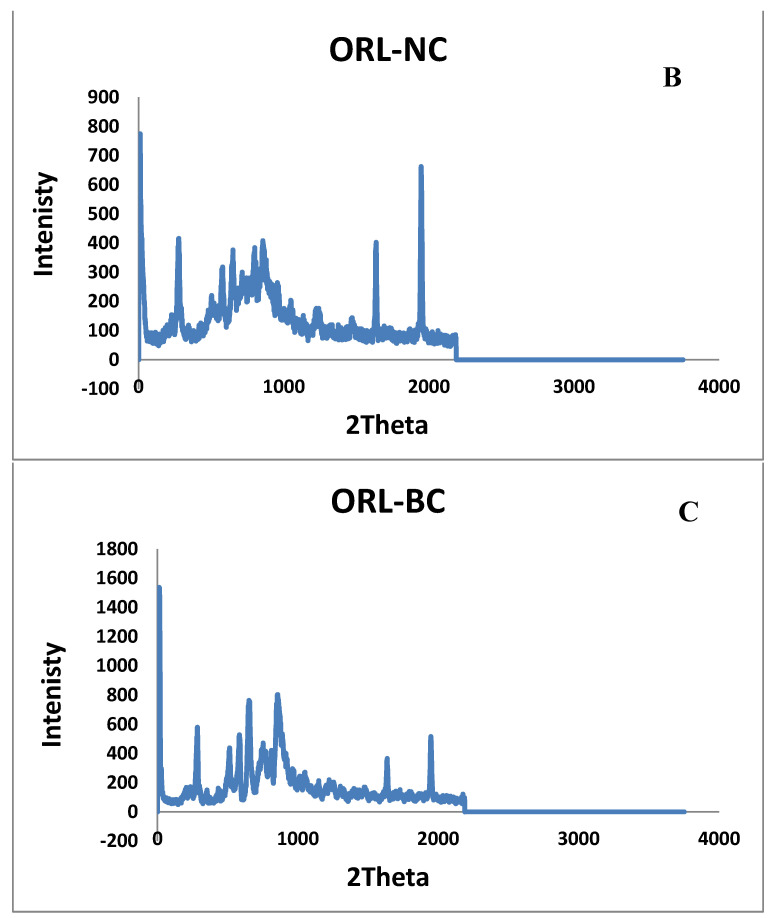
X-ray diffraction.

X-ray diffraction (Figure 4) of ORL, ORL-BC, and ORL-NC was studied to identify any polymorphic alteration that might take place during the manufacture of ORL-NC. The ORL diffractogram showed high crystallinity, with three main distinctive intensity reflection counts at diffraction angles of 2θ: 5.69°, 18.23°, and 19.31°, respectively. These peaks can be seen in the diffractograms of ORL-BC and ORL-NC with almost the same diffraction angles, but there is a substantial decrease in the number and intensity of reflections, especially for ORL-NC.

### 2.6. Dissolution

**Figure 5:**
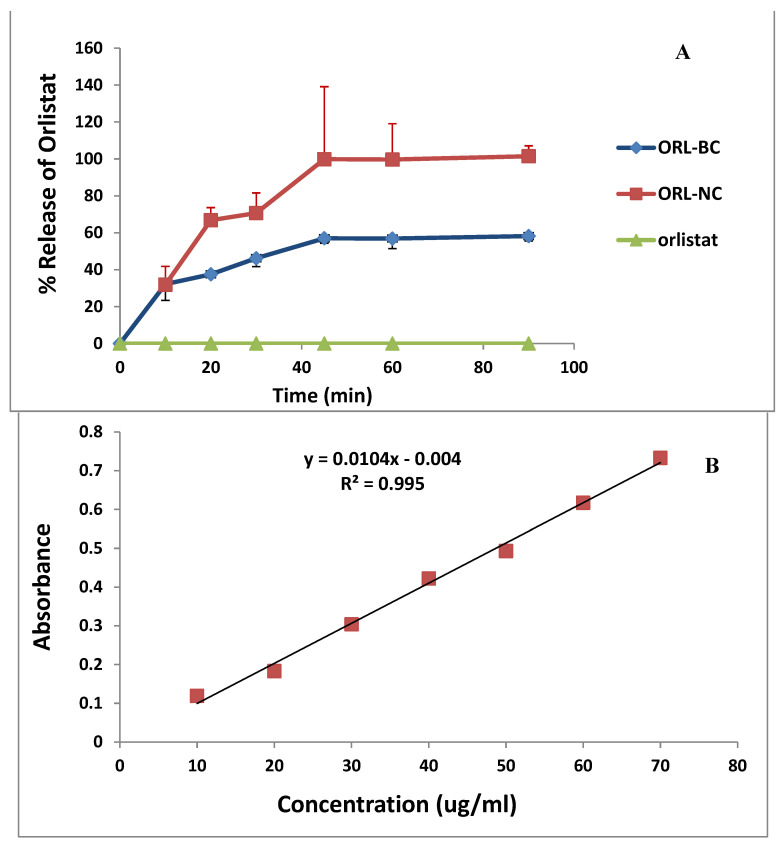
The dissolution profiles of ORL, ORL-NC, and ORL-BC in 0.1 N HCl (**A**) (*n* = 3, mean); the standard curve of ORL in 0.1 N HCl (**B**) (coefficient of variance (CV%) ranged between 2.4 and 10.8 with a % of recovery ranging between 92.74 and 105.4 (*n* = 3)).

The percentages dissolved after 30 min (%Q30) were 70.67 ± 2.18% and 42.79 ± 0.46% for ORL-NC and ORL-BC. However, %Q60 were 100.0 ± 10.5% and 51.60 ± 0.55% for ORL-NC and ORL-BC, respectively. The dissolution efficiency after 30 min (%DE 30 min) was 39.7% and 23.93% for ORL-NC and ORL-BC, respectively, while %DE 60 min was 66.18% and 37.95% for ORL-NC and ORL-BC, respectively. The percentage ratio of DE was calculated using the area under the dissolution curve up to time t, described by the area of the rectangle that corresponds to 100% dissolution at the same time (Table 3 and Figure 5).

### 2.7. Preparation of the ^99m^Tc-Orlistat Nanocrystals Complex

Sodium dithionite was used as a reducing agent during the easy direct technique used to create the ^99m^Tc-orlistat nanocrystal complex. The fundamental advantage of using Na_2_S_2_O_4_ as a reducing agent was that ^99m^TcO_2_ is the only colloidal species formed throughout the reaction [28,29].

### 2.8. Radiochemical Yield Optimization

Examining the aspects that influence the labeling process was crucial for reaching the maximum radiochemical yield. These factors were the quantity of Na_2_S_2_O_4_, the pH of the reaction medium, the quantity of ORL-NC, and the time of the reaction [30]. Table 4 shows the optimum conditions required to obtain maximum radiochemical yield for ^99m^Tc-ORL and ^99m^Tc-ORL-NC. The radiochemical yield of ^99m^Tc-^99m^Tc-ORL-NC was optimized to 84.8% when 1 mg of the substrate was labeled, and the Na_2_S_2_O_4_ quantity was increased to 5 mg at pH 7 for 60 min reaction time. The greatest yield for the plain orlistat was around 98.1% under the following circumstances: 3 mg of substrate and 2 mg of Na_2_S_2_O_4_ for 90 min reaction time and at pH 7.

### 2.9. Serum Stability of the ^99m^Tc-Complex

**Figure 6:**
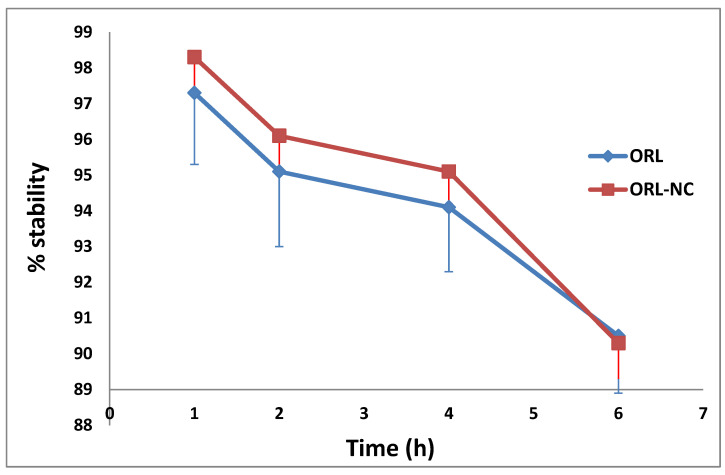
Serum stability of both ^99m^Tc-ORL-NC and ^99m^Tc-ORL complexes’ biodistribution data.

The ^99m^Tc-ORL-NC complex and the ^99m^Tc-ORL complex demonstrated great serum stability for up to 4 h without any visible deterioration, as shown in Figure 6.

### 2.10. Assessment of the Distribution and Bioavailability (Radiography)

Based on little evidence, the absorbed orlistat has a half-life of 1–2 h. Table 5 displays the research findings. The blood uptake of the ^99m^Tc-ORL solution was low throughout all the time points. The activity in bone, muscle, and liver was also extremely low. The intestinal uptake was 6.2 ± 0.5 at 30 min and reached 21.6 ± 1.4 after 2 h. The kidney and urine excretion were low.

**Figure 7:**
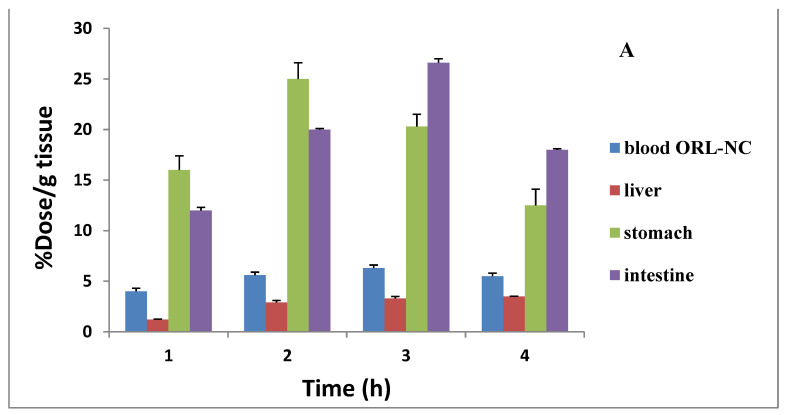

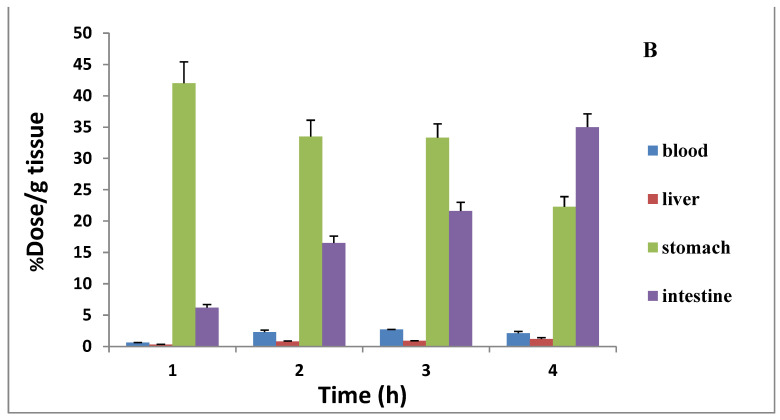
Biodistribution of oral ^99m^Tc-ORL-NC (**A**) and oral ^99m^Tc-ORL (**B**) to blood, liver, stomach, and intestine in normal mice (*n* = 3, mean ± SD). Significant difference at *p* <  0.05. ORL: orlistat and ORL-NC: orlistat nanocrystals.

Table 6 shows the biodistribution of ^99m^Tc-ORL-NC in mice. The mixture was quickly disseminated throughout many organs, including the blood, liver, and lungs. There was a consistently high stomach uptake. Two hours after administration, the intestine showed the maximum uptake (22.6 ± 1.8), and this uptake was twice as much as the uptake of the ^99m^Tc-ORL solution (Figure 7).

A comparison of the extent and rate of drug absorption among products is necessary to assess bioavailability [30]. The absorption rate is generally expressed by $C_{max}$ and $\;T_{max}$, while the area under the curve to the final measurable drug concentration represents the extent of absorption (% dose/gram blood) ($AUC_{0\to 4}$).

**Figure 8:**
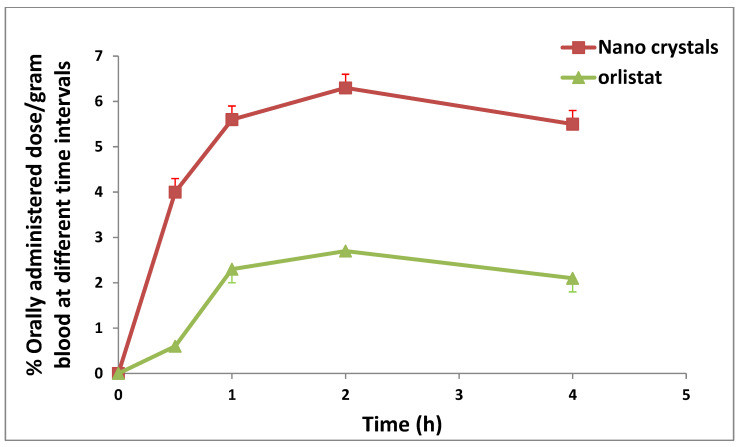
Mean % orally administered ^99m^Tc-ORL and ^99m^Tc-ORL-NC (dose/gram blood) at different time intervals (*n* = 3, mean).

*C*_max_ was 6.3 and 2.7 (% orally administered dose/gram blood) for ORL-NC and ORL, respectively, and $T_{max}$ was achieved straight from the individual plasma concentration vs. the time curve (2 h for both ORL and ORL-NC). The $AUC_{0\to 4}$ was calculated using the linear trapezoidal rule method. The $AUC_{0\to 4}$ was 21.15% and 8.175% orally administered dose/gram blood/h (Table 7 and Figure 8).

The biodistribution of ORL-NC showed a significant difference from ORL, especially in the liver, kidney, and lung (Table 6). On the other hand, tissue biodistribution decreased in both gastric and intestinal tissues of the stomach.

### 2.11. In Vivo Experiments

#### 2.11.1. Effect of ORL and ORL-NC on Serum Liver Function Biomarkers

As shown in Table 8, a solid Ehrlich tumor induced a significant increase (208.4%, 223.62%, and 242.45%, respectively) in the serum content of SGPT, SGOT, and ALP in comparison with the normal control group. In contrast, administration of TAM, ORL, and ORL-NC showed a significant reduction in SGPT serum levels (45.22%, 20.69%, and 37.08%, respectively), in serum SGOT levels (45%, 16.15%, and 34.36%, respectively), and in serum ALP levels (55.12%, 32.43%, and 50.32%, respectively) compared with the SEC control group. In contrast, ORL-NC-treated mice exhibited a significant reduction in serum levels of SGPT, SGOT, and ALP compared with ORL-treated mice (20.65%, 21.72%, and 26.47%, respectively).

Moreover, the solid Ehrlich tumor induced a significant increase (147.06% and 400%) in the serum levels of total and direct bilirubin, respectively, compared with the normal control group. In contrast, administration of TAM, ORL, and ORL-NC showed a significant reduction in the total bilirubin serum level (42.86%, 20.24%, and 32.74%, respectively) and in the direct bilirubin serum level (50%, 27.78%, and 38.89%, respectively) compared with the SEC control group. In contrast, the ORL-NC-treated mice showed a significant reduction (15.67% and 15.38%) in the serum levels of total and direct bilirubin, respectively, compared with the ORL-treated mice.

#### 2.11.2. Effect of ORL and ORL-NC Protected the Hepatic Tissue of Mice from SEC-Induced Histopathological Changes

**Figure 9:**
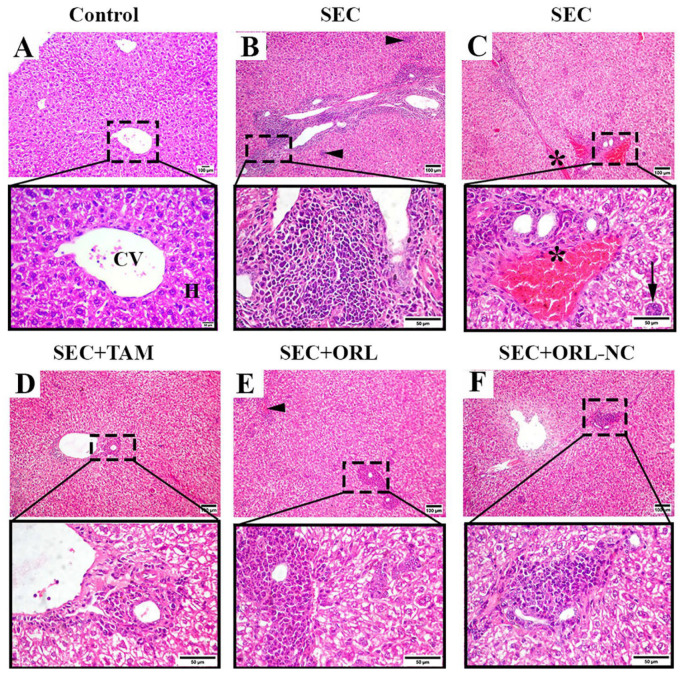
Photomicrographs of hepatic sections of solid Ehrlich carcinoma (SEC)-bearing mice and/or treated with tamoxifen (TAM), ORL (orlistat), and ORL-NC (orlistat nanocrystals) stained with H&E. (**A**) Control group showing typical central vein (CV) with radiating hepatic plates. (**B**,**C**) Hepatic section of untreated tumorized mice showing pleomorphic cells admixed with severe inflammatory cell infiltration, multifocal areas of hepatocellular necrosis (arrowheads), congested vessels (*), and giant cells (arrow). (**D**) TAM-treated group showing moderate hepatic vacuolation and minute inflammatory infiltrates without tumor metastasis. (**E**) ORL-treated group showing congested vessel (arrowhead) and moderate inflammatory infiltration with mild tumor cell invasion. (**F**) ORL-NC-treated group showing hepatocellular vacuolation with mild inflammatory infiltrates without tumor cell invasion.

Microscopic examination of H&E-stained hepatic sections from the control mice presented a normal hepatic structure with a normal central vein, radiating hepatic cord and portal areas (Figure 9A). Hepatic sections of untreated SEC-bearing mice showed pleomorphic cells with hyperchromatic nuclei supposed to be SEC tumor cells admixed with severe inflammatory cell infiltration, multifocal areas of hepatocellular necrosis (Figure 9B), congested vessels, and giant cell formation (Figure 9C). Additionally, hepatocellular vacuolation with nuclear enlargement and binucleation was also observed. Conversely, hepatic slices of TAM-treated mice displayed moderate vacuolated hepatocytes with mild cellular infiltration and without tumor invasion (Figure 9D). Liver sections of SEC-bearing mice treated with ORL exposed hepatocellular vacuolation and necrosis and moderate cellular infiltrates admixed with few cancerous cells (Figure 9E). Liver tissue sections of SEC-bearing mice correlated with ORL-NC showed moderate vacuolar degeneration and mild-to-moderate cellular infiltrates without tumor cell invasion (Figure 9F).

#### 2.11.3. Effect of ORL and ORL-NC Reduced SEC-Induced Increased ER-α and Ki-67

**Figure 10:**
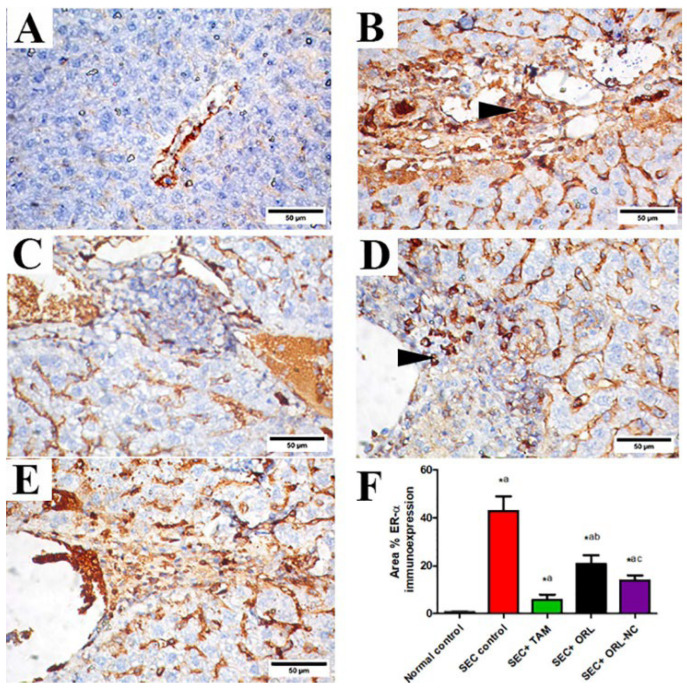
Photomicrographs of hepatic sections of solid Ehrlich carcinoma (SEC)-bearing mice and/or treated with tamoxifen (TAM), ORL (orlistat), and ORL-NC (orlistat nanocrystals) stained with estrogen receptor (ER-α). (**A**) Control group. (**B**) Hepatic section of untreated tumorized mice showing strong positive ER-α expression (arrowhead). (**C**) TAM-treated group showing negative ER-α expression. (**D**) ORL-treated group showing moderate ER-α expression (arrowhead). (**E**) ORL-NC-treated group showing nearly negative ER-α protein expression. (**F**) Semiquantitative analysis of ER-α protein expression in different experimental groups. Scale bar = 50 µm. Bars show mean ± SD (*n* = 6/group), significant difference vs. ^a^ SEC group, ^b^ SEC+TAM group, ^c^ SEC+ORL group, each group significantly different at *p* < 0.05.

Liver tissue sections of the control group revealed negative ER-α immunoexpression (Figure 10A). Liver tissue sections of SEC-treated mice showed strong ER-α immunostaining (Figure 10B). Liver sections of SEC-treated mice co-treated with TAM showed nearly negative ER-α immunostaining (Figure 10C), moderate expression in the ORL-treated group (Figure 10D), and mild to nearly negative expression in the ORL-NC-treated group (Figure 10E). Semiquantitative analysis of ER-α revealed that compared with the control mice, the untreated SEC-bearing mice showed a marked increment in the area % of ER-α protein expression. Conversely, in SEC-bearing mice co-treated with TAM and ORL, ORL-NC presented a significant decline in the area % of ER-α protein expression, respectively, in comparison with untreated SEC-bearing mice with a more pronounced effect in TAM than the ORL-NC-treated group (Figure 10F).

**Figure 11:**
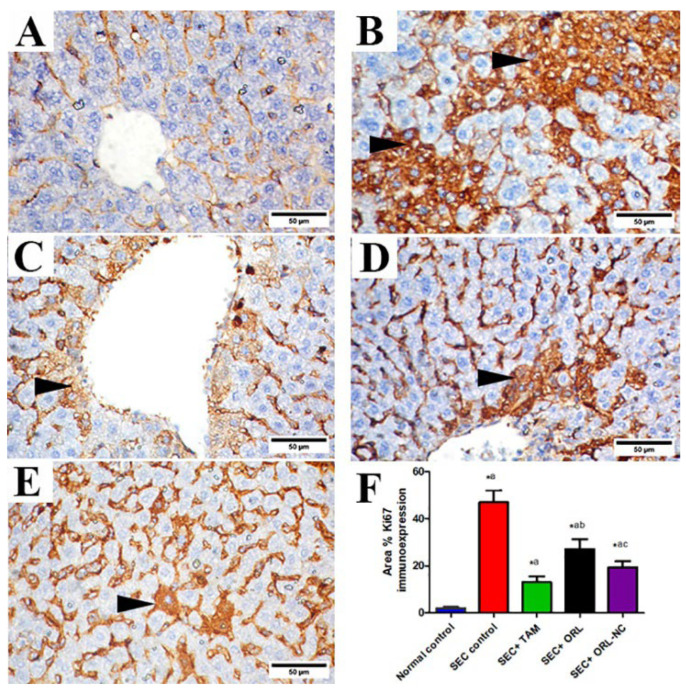
Photomicrographs of hepatic sections of solid Ehrlich carcinoma (SEC)-bearing mice and/or treated with tamoxifen (TAM), ORL (orlistat), and ORL-NC (orlistat nanocrystals) stained with Ki-67. (**A**) Control group. (**B**) Hepatic section of untreated tumorized mice showing strong positive Ki-67 cytoplasmic expression (arrowheads). (**C**) TAM-treated group showing faint Ki-67 expression (arrowhead). (**D**) ORL-treated group showing moderate Ki-67 cytoplasmic expression (arrowhead). (**E**) ORL-NC-treated group showing mild Ki-67 protein expression (arrowhead). (**F**) Semiquantitative analysis of Ki-67 protein expression in different experimental groups. Scale bar = 50 µm. Bars showed mean ± SD (*n* = 6/group), significant difference vs. ^a^ SEC group, ^b^ SEC+TAM group, ^c^ SEC+ORL group, each group significantly different at *p* < 0.05.

Moreover, the control group’s liver tissue sections showed negative or mild Ki-67 cytoplasmic immunoexpression (Figure 11A). Liver tissue sections of SEC-treated mice showed strong Ki-67 cytoplasmic immunostaining (Figure 11B). Liver sections of SEC-bearing mice treated with TAM (Figure 11C) and ORL-NC (Figure 11E) showed faint to nearly negative cytoplasmic immunostaining of Ki-67 and moderate expression in the ORL-treated group (Figure 11D). Semiquantitative analysis of Ki-67 revealed that compared with the control mice, the untreated SEC-bearing mice showed a marked increment in the area % of Ki-67 protein expression. Conversely, SEC-bearing mice co-treated with TAM, ORL, and ORL-NC displayed a significant reduction in the area % of Ki-67 protein expression compared with untreated SEC-bearing mice, with a more pronounced impact in the TAM-treated mice than the ORL-NC-treated mice (Figure 11F).

## 3. Discussion

The effective formulation of medications depends on several factors, including solubility, stability at room temperature, and compatibility with solvent, excipient, and photostability. Currently, approximately 40% of newly created chemical entities resulting from drug development initiatives are lipophilic or poorly soluble in water substances. The challenges posed by the previously discussed methods can be resolved via nanotechnology. The study of science and engineering at the nanoscale, or 10–9 m, is known as nanotechnology. Techniques such as bottom-up technology are used to transfer the drug microparticles or micronized drug powder to the drug nanoparticles [31].

ORL-NC was prepared using the liquid anti-solvent precipitation method (bottom-up technology) without the use of expensive polymers or complicated procedures to enhance its solubility. Employing the radiolabeling technique provides this study with a superior advantage not only for tracing ORL in blood, as in the traditional analytical method (even the HPLC/MS/MS technique), but also for tracing the drug in different body organs and selecting the liver based on the results of biodistribution. The results again confirmed that ORL-NC enhances the solubility and, consequently, the bioavailability and tissue distribution to the liver (passive targeting), leading to an increase in the anticancer role of ORL and ORL-NC against SEC-induced liver damage in comparison with conventional tamoxifen treatment.

Saturation concentration is a solubility measure of a substance in a specific solvent. The saturation point of a substance can be exceeded under certain conditions, resulting in a so-called supersaturated solution [3]. The dissolving rate increases by increasing the surface area (Noyes–Whitney equation). In addition to the nature of the drug molecule, the dissolving medium, and the temperature, the apparent saturation solubility (Cs) of pharmaceuticals also depends on the particle size when it is below 1 µm [32]. The rate of dissolution is further accelerated by an increase in apparent saturation solubility. The presence of Tween 80 contributed to better particle stability in the nanosized range, as evidenced by the higher solubility of ORL-NC compared with ORL-BC in both aqueous and 0.1 N HCl.

The stabilizer is essential for decreasing the size of the particles and preserving the stability of nanosuspensions. Stabilizers can create a dense hydrophilic layer around hydrophobic particles, offer steric hindrance and steep repulsions between the particles (steric stabilization), and spontaneously adsorb on and cover the newly formed particle surface to reduce the free energy of the system and the interfacial (surface) tension of the particles. The steric barriers provided by the stabilizer are the key factors preventing the drug NPs from coming into contact with one another. The more stable nanosuspensions that are produced have greater barrier energy.

The DSC thermograms of ORL, ORL-NC, and the control crystals (ORL-BC) indicate slight changes in the endothermic melting point. The drug was reduced to a nanocrystalline state, as shown by a minor change in the melting point. As a result of the high rate of exposure, most medications were transformed into their nanocrystalline form [27].

The XRD results coincide with the previous observations, revealing peaks that indicate a substantial decrease in the number of reflections of ORL-NC compared with pure ORL powder. Particle size reduction was likely the reason for the lower peak intensity. Smaller-sized drugs typically have improved bioavailability and dissolving rates [33]. The results of the particle size analysis, SEM, DSC, XRD, and the dissolution profile of ORL-NC all provide strong evidence for enhancing ORL solubility and bioavailability and, consequently, improving therapeutic efficiency after oral administration.

Due to the lack of a strong chromosphere of ORL and to study the new tissue distribution of ORL-NC, the ^99m^Tc-ORL and ^99m^Tc-ORL-NC complex was prepared using sodium dithionite as a reducing agent. The main benefit of using Na_2_S_2_O_4_ as a reducing agent is that no colloidal species other than ^99m^TcO_2_ are produced in the reaction [3,4,5,6,7,8,9,10,11,12,13,14,15,16,17,18,19,20,21,22,23,24,25,26,27,28]. Radiochemical yield optimization was investigated to determine the conditions required to obtain maximum radiolabeling yield by researching the variables influencing the labeling process. These factors are the quantity of ORL-NC, the quantity of Na_2_S_2_O_4_, the pH of the reaction medium, and the reaction time. The result illustrated that the radiochemical yield depends on the amount of the ligand (quantity of ^99m^Tc-complex). The radiochemical yield was increased by increasing the amount of ligand (maximum yield 84.4% at 1 mg ligand); after this value, it showed a very slight decrease [30].

The radiolabeling technique is a unique method for tracing ORL; this method not only allows for the investigation of the relative bioavailability and extent of drug concentration in the blood, as per the conventional analytical method, but also provides much more detail about tissue distribution in different body organs.

One of the key components in the radiolabeling process is sodium dithionite, which is used to convert the pertechnetate ion (^99m^TcO_4_^−^) from its heptavalent state to the chemically reactive reduced states of the ^99m^Tc^+3^ and ^99m^Tc^+4^ species [34]. The results of this study illustrated that 0.5 mg of Na_2_S_2_O_4_ is insufficient to completely reduce all the pertechnetate ions, resulting in an inferior ^99m^Tc-ORL-NC yield and a very high percentage of free pertechnetate. The radiochemical yield of ^99m^Tc-^99m^Tc-ORL-NC was increased to 84.8% when the Na_2_S_2_O_4_ quantity was increased to 5 mg. Concerning pH effects, the yield dropped with both acidification and alkalization; this result can be explained by the partial hydrolysis of the ^99m^Tc-complex, which is caused by greater H^+^ and OH^−^ concentrations at acidic and alkaline pH values, respectively [34]. The reaction was conducted at various intervals to identify the most practical time for making the ^99m^Tc-ORL-NC complex. The result demonstrated how the labeling yield rises progressively as the reaction time increases until it reaches its maximum value at 60 min and does not significantly change over this value [35]. Nevertheless, the most significant yield for the plain ORL was around 98.1% under the following circumstances: 3 mg substrate and 2 mg NaBH_4_ for 90 min and at pH 7. The ^99m^Tc-ORL-NC and 99mTc-ORL complexes demonstrated high serum stability for up to 4 h without any visible deterioration.

In all biodistributions of ^99m^Tc radiotracers, the thyroid functions as a suggestive organ of the complex stability in the biodistribution, which makes the complex preferentially reoxidized in vivo to liberate ^99m^TcO_4_^−^ and, consequently, it may exhibit significant activity. Accordingly, this case demonstrated that the technetium complex is stable in vivo because less than 5% of the administered dose was found in the thyroid [36]. Based on little evidence, the absorbed ORL has a half-life of one to two hours. The blood uptake of the ^99m^Tc-ORL solution was low throughout all the time points. The activity in bone, muscle, and liver was extremely low. The kidney and urine excretion were low because ORL and its metabolites are subject to biliary excretion.

On the other hand, the biodistribution of ^99m^Tc-ORL-NC in mice is rapidly disseminated throughout many organs, including the blood, liver, and lungs. There is consistently high stomach uptake. At two hours after administration, the intestine showed the maximum uptake (22.6 ± 1.8), which was twice as much as the uptake of the ^99m^Tc-ORL solution.

Analyzing bioavailability has focused on comparing how quickly and how much a drug is absorbed from different formulations [37]. After oral administration, nano- and microparticles can follow at least three possible elimination pathways: (i) capture by gut-associated lymphoid tissue, (ii) mucoadhesion, and (iii) direct fecal evacuation during gastrointestinal transit [38]. The mucous gel layer is a porous framework for NPs. The NPs have a rapid depth penetration and can directly contact the mucosal network. The linear rise in the adsorption isotherm with particle concentration was observed. As a result of the particle size being too big to pass through the mucosal gel layer, the mucosal layer for microparticles looks more like a smooth surface than a porous one. Accordingly, NPs improve the ability of adhesiveness and reduce the adsorption time. The contact time and retention time with the membrane of the gastrointestinal tract (GIT) are prolonged by the enhanced adhesiveness of NPs [13].

The concentration gradient then increases as the diffusion distance of the NPs to the GIT membrane shortens. The NCs can effectively increase bioavailability; using NCs may result in a larger $C_{max}$, a quicker commencement of action, and a quicker dissolution in the constrained absorption window. The biodistribution of ORL-NC shows a significant difference from ORL, especially in the liver, kidney, and lung. However, the tissue biodistribution decreases in both gastric (stomach) and intestinal tissues. These results suggest an enhanced tissue uptake and, consequently, a more efficient anticancer effect of ORL in these tissues, in addition to enhanced aqueous solubility, which enables ORL formulation as an intravenous aqueous solution and eliminates the danger of embolism.

The shape of the NP has a significant effect on its ability to penetrate enterocytes. The NCs showed superior transcellular absorption compared with the spherical and flaky-shaped NPs [14]. The Golgi/plasma membrane and endoplasmic reticulum/Golgi were reported to be involved in the transcellular absorption of NCs. Additionally, NCs are characterized by strong bioadhesion, elevated saturation solubility, and quick dissolution velocity. Long-rod NCs exhibited bottom-most liver distribution renal excretion and in vivo biodegradation in comparison with spherically shaped NPs [14].

Meanwhile, solid Ehrlich carcinoma is undifferentiated, develops quickly, and is more sensitive to chemotherapies; it is analogous to human cancer [39,40]. The growth of a tumor might harm the essential organs, especially the liver. Herein, we aimed to identify the anticancer role of ORL and ORL-NC against SEC-induced liver damage using standard tamoxifen chemotherapy.

Our results showed that SEC caused hepatic dysfunction, as evidenced by elevated serum ALT, AST, ALP activity, and bilirubin (total and direct), consistent with the findings of many other studies [41,42,43]. The histopathological examination of hepatic sections in untreated SEC-bearing mice revealed solid sheets of carcinoma cells with marked inflammatory cell infiltrates, giant cells, hepatocellular vacuolation, and necrosis. These results parallel previous investigations indicating the proliferation and metastasis of carcinoma cells into many internal organs [40]. The inflammatory cellular infiltrates responsible for hepatic inflammation progression may be attributed to mitochondrial damage [44,45,46] or the harmful effects of tumor angiogenesis caused by proangiogenic factor secretion [47].

Moreover, 70–75% of breast cancer cells express ER-α, which is related to the extent of tumor estrogen dependence required for tumor development and survival [47]. Our study revealed significant ER-α protein expression in the liver. Earlier studies showed ER-α immunoexpression in SEC thigh muscle [48,49]. Nevertheless, to the best of our knowledge, we are the first to report the presence of positive ER-α immunostained metastatic cancerous cells in the liver. In the same context, Ki-67 is a proliferating nuclear antigen expressed in replicating cells across all cell cycle phases except G0, with G2/M showing the highest expression levels [50]. Our study showed significant Ki-67 immunoexpression in the liver of SEC-bearing mice, which is consistent with earlier studies [51,52].

**Figure 12:**
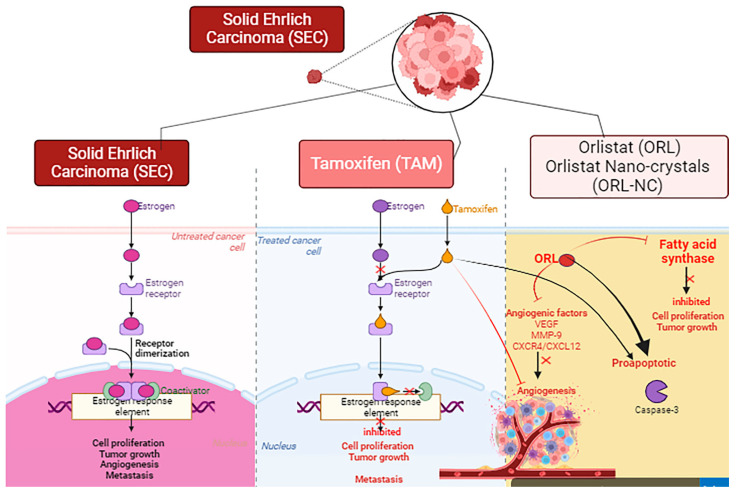
A model of the mechanistic action of the antitumoral activity of tamoxifen (TAM), orlistat (ORL), and orlistat nanocrystals (ORL-NC) in breast cancer. Created with BioRender.com.

In addition, TAM-, ORL-, and ORL-NC-treated groups showed a decrease in liver function markers. In addition, histopathological examination of hepatic sections depicted similar lesions, but these were less widespread and severe. Several studies have reported the antitumor activity of TAM against breast cancer by blocking estrogen receptors, inhibiting angiogenesis, and increasing apoptosis [53,54]. Our study is parallel to previous reports that showed the antitumor activity of ORL [7,8,9,10,11,12,13,14,15,16,17,18,19,20,21,22,23,24,25,26,27,28,29,30,31,32,33,34,35,36,37,38,39,40,41,42,43,44,45,46,47,48,49,50,51,52,53,54,55]; however, only one study reported the therapeutic efficiency of orlistat nanoformulation against triple-negative breast cancer [12]. It has been shown that the antitumoral activity of ORL is linked with its ability to block fatty acid synthase (FAS) activity, which subsequently inhibits the proliferation of Her2/neu breast cancer cells [2]. In another study, orlistat demonstrated notable efficacy in altering cancer cell metabolism through its antiproliferative, proapoptotic, antiangiogenic, antimetastatic, and hypolipidemic properties. ORL inhibits angiogenesis (VEGF, MMP-9, and CXCR4/CXCL12) and fatty acid synthesis (ACLY, ACC, and FASN) genes and protein expression with the induction of apoptotic genes in different breast cancer cell lines [2] (Figure 12).

Tetrahydrolipstatin, the generic name for the FDA-approved drug ORL (Xenical), is now marketed as an over-the-counter weight loss aid and is used to assist people with diabetes in losing weight [56]. Additionally, several studies have demonstrated its anticancer effect by suppressing fatty acid production [55]. ORL has a lipogenic effect by suppressing FAS, an oncogenic antigen-519 that is elevated in more than 50% of breast tumors and associated with a poor prognosis. The expression of FAS in tumors is not influenced by dietary signals due to the distinct mechanism that regulates FAS expression in cancer cells compared with normal tissues. Despite similar downstream pathways, the signal transduction pathways that regulate FAS expression in healthy and malignant cells remain distinct. FAS expression becomes more specific to tumors because it does not affect the liver and adipose tissues, which are often involved with FAS [57].

However, ORL has a relatively poor bioavailability (1%) due to its hydrophobic nature, which causes significant issues [58]; accordingly, NPs have been investigated in many studies as a potential tool for drug delivery [59,60]. Biodegradable polymeric NPs, which offer a vehicle for prolonged drug delivery and release, are less harmful since they are likely to congregate in the metabolized body [61,62]. The science of nanomedicine has made tremendous strides recently, and numerous NP drug delivery systems are currently undergoing various stages of clinical development [63].

Additionally, treatments with TAM and ORL-NC showed nearly negative ER-α immunoexpression, indicating their effect against cancer metastasis. Further, ORL treatment in our investigation was shown to decrease ER-α protein expression. Therefore, the antitumor activities of TAM, ORL, and ORL-NC against SEC-bearing mice are associated with their ability to inhibit the metastasis of ER-α immunopositive cells to the liver. To the best of our knowledge, this is the first study to show the antitumor activity of TAM, ORL, and ORL-NC via the inhibition of ER-positive breast cancer cells in the liver. In addition, treatments with TAM, ORL, and ORL-NC significantly reduced Ki-67 protein expression compared with untreated SEC-bearing mice. Consequently, the antitumor activities of TAM, ORL, and ORL-NC against SEC-bearing mice are related to their ability to suppress the proliferation marker Ki-67 immunoexpression.

Finally, the results of this study show a successful technique to develop both the solubility and bioavailability of a challenging, low-soluble drug and a promising anticancer drug candidate.

## 4. Materials and Methods

### 4.1. Drugs and Chemicals

The ORL was kindly provided by EVA PHARMA. Tween 80, ethanol, and hydrochloric acid (El-Nasr Pharmaceutical Chemical Co., Obour, Egypt) were used. Deionized water was freshly collected (Stakpure, Waters, Milford, MA, USA). All additional chemicals and reagents that were highly analytically pure were bought from the Merck Company (Darmstadt, Germany) and used directly. A ^99m^Mo/^99m^Tc generator was provided by the Egyptian Atomic Energy Authority’s radioisotopes production facility (RPF), and the Egyptian Second Research Reactor (ETRR-2) was used to elute sodium pertechnetate (Na [^99m^Tc] TcO4). Tamoxifen was bought from the Egyptian company Techno Pharmaceutical Co. (Alexandria, Egypt).

### 4.2. Preparation of ORL Nanocrystals

ORL nanocrystals (ORL-NC) were prepared using the solvent change method [64,65]. Four grams of ORL powder was dissolved in 10 mL of ethanol. The resultant mixture was promptly injected (using a 10 mL syringe (22G)) into 100 mL of 1% Tween 80 deionized aqueous solution. Continued stirring (300 rpm/37 °C) of the obtained dispersion (Stuart, Calibre Scientific, Holland, OH, USA) was maintained for 30 min and then subjected to sonication (50 s on and 2 s off, Ampl 50%, energy 203 J) for 5 min (SONIC Vibracell TM, USA (86916W-09-15)). All procedures were repeated to fabricate ORL-BC except for the addition of a stabilizer (Tween 80) to deionized water. The crystals were centrifuged (3000 rpm for 20 min, 37 °C) (Centurion Scientific, Chichester, UK). The crystals were separated and rinsed twice (20 mL of deionized water), transferred into an amber glass container, and then subjected to freeze drying (Christ Benchtop Freeze dryer, Osterode am Harz, Germany).

### 4.3. Characterization of ORL Crystals

#### 4.3.1. Saturation Solubility

In airtight screw-capped amber bottles, the saturation solubility of ORL, ORL-NC, and ORL-BC crystals was examined by adding a large excess of samples to 10 mL of deionized water and 0.1 N HCl. The bottles were kept on a magnetic stirrer (Stuart, Calibre Scientific, USA) for 48 h at 100 rpm and 25 °C to achieve equilibrium, and the equilibrated samples were centrifuged at 3000 rpm for 5 min. Supernatants were divided into aliquots and filtered via 0.22 µm syringe membrane filters, and the filtrate was spectrophotometrically measured at 209 nm [66,67]. Each sample was examined in triplicate, and ORL concentration was calculated according to the following equation (R2 = 0.995):

ORL conc. = (0.0053 ± 0.0034) absorbance ± (0.151 ± 0.0625)

#### 4.3.2. Particle Size Analysis

The mean particle size and zeta potential (Malvern Panaltyical, Malvern, UK, software version 3.1.0.64) were analyzed following the dispersion of ORL-NC and ORL-BC to produce saturated aqueous solutions at 25 ± 0.5 °C.

#### 4.3.3. Scanning Electron Microscopy (SEM)

ORL-NC topography was monitored using SEM (Thermo Scientific’s Quattro scanning electron microscope). The samples were coated with gold using a sputter coater (SPI-Module).

#### 4.3.4. Differential Scanning Calorimetry (DSC)

Using thermal equipment for analysis (PerkinElmer thermal analysis, Shelton, CT, USA), DSC was conducted on the ORL powder, ORT-NC, and ORL-BC to determine any potential changes in the physical state of ORL in the produced crystals. The samples (10 mg) were heated in an aluminum (30 to 220 °C) pan with nitrogen gas at a constant rate of 5 °C/min. An identical empty pan was used as a guide.

#### 4.3.5. X-ray Powder Diffraction

X-ray diffraction patterns of the received drug, OLR-BC, and ORL-NC were collected (X’Pert-PRO Diffractometer, PANalytical, Almelo, The Netherlands) to investigate ORL’s physical state using a cupper as the tube anode. The diffractograms were captured at room temperature (40 kV voltage, 30 mA current, steps of 0.020 of (°20), and scan speed 8 (deg/min), sampling pitch 0.02 (deg), and counting time of 0.15 s).

#### 4.3.6. In Vitro Dissolution Study

A type II dissolve apparatus (Copley Scientific, UK) was used to compare the dissolving behaviors of ORL-NC, ORL powder, and ORL-BC crystals. The dissolution tests were carried out in triplicate. The rotation speed of the paddles was 75 rpm. An amount of 900 mL of 0.1 N HCl (37 °C) was used [6]. Precisely weighed samples equivalent to 60 mg of ORL were added to the dissolution media. Samples (5 mL) were filtered using a 0.2 μm syringe filter. Withdrawn samples were compensated with a fresh medium.

### 4.4. Bioavailability and Tissue Distribution Study

Tissue distribution and relative bioavailability were designed to investigate the difference between ORL-NC and the received ORL powder with the aid of radiolabeling as a concentration tracing technique.

#### 4.4.1. Radiolabeling of ORL Using Technetium-99m

In an evacuated vial, a mixture of 3 mg of ORL in 0.3 mL of DMSO was mixed with 2 mg of sodium borohydride (NaBH_4_) in pH 7 medium at room temperature, and 20 mL of a freshly eluted pertechnetate solution (^99m^TcO_4_^−^) (~60 MBq) was added to this combination. The reaction mixture was then maintained for 90 min. The same method was conducted with various concentrations of ORL to evaluate the parameters impacting labeling process yields (0.25–5 mg) and NaBH_4_ (0.25–5 mg) at various pH levels (3–10). Each experiment was repeated three times, and other variables remained unchanged.

#### 4.4.2. Assessment of Radiochemical Yield

Acetone was used as the initial mobile phase in the development of ascending paper chromatography (APC) using strips of Whatman No. 1 chromatography paper (12 cm length and 1 cm width) (Whatman International Ltd., Maidstone, Kent, UK). The radiochemical yield (RCY) of ^99m^Tc-ORL-NC was evaluated using acetone as a mobile phase to determine the free ^99m^TcO_4_^−^ percent. One spot of the reaction was applied 2 cm away from the base of the strip, which then developed in a jar containing a few drops of acetone. After developing, the strip was dried and sliced into 1 cm pieces and then counted using a NaI (Tl) γ-ray scintillation counter. The ^99m^Tc-ORL-NC complex was calculated using the formula below.

Radiopharmaceutical purity (RCP):

Pertechnetate ^99m^TcO_4_^−^ (%) = (activity at Rf = 1.0)∕(total activity) × 100

Radiolabeled ^99m^Tc−complex (%) = (100 − free ^99m^TcO_4_^−^) × 100

#### 4.4.3. Serum Stability

To assess the in vitro stability, 1.8 mL of freshly collected human blood and 0.2 mL of ^99m^Tc-ORL-NC were combined. After the combination was incubated at 25 °C for 24 h, 50 µL of the mixture was removed at various intervals and examined on chromatography paper [68].

#### 4.4.4. Assay for Biodistribution

This study was authorized by the Egyptian Atomic Energy Authority’s Labelled Compounds Department’s animal ethics committee and followed the regulations established by Cairo University’s Faculty of Pharmacy (PT 4.2.2) and its animal ethics committee’s guidelines. This experiment was conducted using 24 mice divided randomly into two equal groups: one received a ^99m^Tc-ORL solution, and the other received oral ^99m^Tc-ORL-NC. Each group was divided into four further subgroups, each representing certain time intervals (30, 60, 120, and 240 min). All mice were housed, received the same diet, and were given free access to water overnight during fasting for 12 h. Biodistribution of the oral ^99m^Tc-ORL solution and the oral ^99m^Tc-ORL-NC was performed. The percentage of orally administered dose/gram tissue was calculated in the following organs: blood, bone, muscle, brain, thyroid gland, liver, intestine, stomach, heart, lung, spleen, kidney, and urine [69].

As a function of time, 30, 60, 120, and 240 min after oral administration, the biodistribution of the ^99m^Tc-ORL and ^99m^Tc-ORL-NC complex in mouse organs and fluids was examined. Each mouse received an orally administered 0.2 mL of ^99m^Tc-complex (20 MBq). The animals were euthanized, and then their internal organs were removed, cleaned with saline, and counted using an X-ray scintillation counter. Weights for blood, bone, and muscle were determined to be 7%, 10%, and 40% of the total body weight, respectively [69]. The outcomes are presented as a percentage dosage per gram of organ/fluid.

### 4.5. In Vivo Experiments

#### 4.5.1. Animals

Thirty mature female Swiss albino mice weighing 22–25 g were obtained from the National Institute of Ophthalmology in Giza, Egypt. They were fed on standard pellet chow produced by the EL-Nasr Chemical Company in Cairo, Egypt, and had unrestricted access to water. Before the trial, the animals were kept in the same settings for a week to acclimatize. The research ethics committee of Tanta University’s Faculty of Pharmacy approved this study, and it adhered to the guidelines of the Council for International Organizations of Medical Sciences (CIOMS) (protocol code: TP/RE/8/23p-0043).

#### 4.5.2. Induction of the Solid Ehrlich Tumor

The Ehrlich ascites carcinoma (EAC) cell line was acquired from the Pharmacology and Experimental Oncology Unit of the National Cancer Institute (NCI), Cairo University, Egypt. Both solid and ascetic EAC cell growth was possible. The initial tumor for EAC cells was a spontaneous mouse breast cancer, and these cells are of mammary origin. Within ten days, Ehrlich tumor cells appeared in the ascetic fluid and were collected via IP puncture using a sterile syringe and dilution. The cells were counted using a Neubauer Hemocytometer (Sigma-Aldrich, St. Louis, MO, USA) [70]. The cells were determined to represent more than 99% of viability using the trypan blue dye exclusion technique [71]. Then, an SEC model in female Swiss albino mice was established. Briefly, 0.2 mL of live EAC cells (5 × 10^6^/mL) were injected subcutaneously in the left thigh of the lower limb and left for 12 days. A 14-day treatment cycle with several medications was started on the 12th day after injecting the EAC cells [72,73].

#### 4.5.3. Experimental Design

The mice were randomly allocated into five groups (six rats each): (i) normal control group; (ii) SEC control vehicle: 0.2 mL (5 × 10^6^/mL) of live EAC cells were injected subcutaneously into the thigh of the lower limb of each mouse and distilled water and ethanol were administered; (iii) SEC+TAM: mice were injected with tamoxifen (10 mg/kg bd. wt., IP) dissolved in distilled water once daily for 14 days [74]; (iv) SEC+ORL: mice were injected with ORL (240 mg/kg bd. wt., IP) dissolved in 10% ethanol once daily for 14 days (Saleh et al., 2019); (v) SEC+ORL-NC: SEC mice were injected with ORL-NC (240 mg/kg bd. wt., IP) dissolved in 10% ethanol once daily for 14 days [8].

##### Blood and Tissue Collection

Under light anesthesia by isoflurane, blood was collected using a jugular vein puncture and placed in clean and dry test tubes at the end of the experiment on day 26. The blood was allowed to coagulate for 15 min at 4 °C and was then centrifuged (BHG, Hermle Z 230) at 3000 rpm for 15 min. For biochemical examination, a serum sample was obtained and stored below 20 °C; it was then tested for alkaline phosphatase, glutamic pyruvic transaminase, glutamic oxaloacetic transaminase, and total and direct bilirubin levels. The mice were sacrificed by cervical dislocation, and the liver tissues were removed.

##### Liver Enzyme Measurement

Serum glutamic pyruvic transaminase (Cat. No. MBS264717), glutamic oxaloacetic transaminase (Cat. No. MBS2019147), and alkaline phosphatase (Cat. No. MBS725505) activities, as well as total (Cat. No. MBS756027) and direct bilirubin (Cat. No. MBS109212), were measured using ELISA kits (My Bio-Source Company, San Diego, CA, USA) per the manufacturer’s instructions, and the enzyme activity was expressed for total and direct bilirubin as IU/L and mg/dL, respectively.

##### Histopathological Assessments

Liver samples were carefully removed, fixed right away in a 10% phosphate-buffered formalin solution, and processed using the standard paraffin embedding method. Then, the samples were sectioned at a thickness of 4 µm and stained with Mayer’s hematoxylin and eosin (H&E) stain for histopathological examination [75].

##### Immunohistochemical Protein Assay

Liver sections with 4 µm thickness were placed on positively charged slides and then deparaffinized, rehydrated, and microwave-treated (10 mM sodium citrate buffer) for retrieval of antigen. In order to suppress endogenous peroxidase and non-specific reactions, slides were then treated for 30 min with 3% hydrogen peroxide in PBS. Following this, 10% regular blocking serum was applied to the slides at room temperature for 60 min. The slides were incubated overnight in a humidified chamber at 4 °C with mouse monoclonal primary antibody against Er-α (Estrogen Receptor alpha, Catalog # (H226): sc-53493, 1:50 dilution, Santa Cruz, CA, USA) and Ki-67 (Catalog # sc-23900, 1:50 dilution, Santa Cruz, CA, USA), and then with horseradish peroxidase-conjugated secondary antibody for 30 min at 37 °C. The slides were washed with phosphate buffer saline three times following each step. The sections were incubated with 3,3′-diaminobenzidine tetrahydrochloride reagent for 3 min. Eventually, the slides were rinsed with distilled water, counterstained with Mayer’s hematoxylin solution, and mounted using DPX. The slides were examined microscopically, and digital micrographs were captured using a digital camera connected to a microscope (Olympus CX21, Shinjuku-ku, Tokyo, Japan). Using ImageJ analysis software (Fiji Image J, 1.51n, NIH, Bethesda, MD, USA), the inverse mean density of immunopositive regions for Er-α and Ki-67 was measured in 10 randomly selected micrographs from each mouse in each group. It was then reported as a percentage of positive area per mm^2^ [76].

### 4.6. Analysis of the Data

The findings of % dose/gram tissues and biodistribution were compared using the Student’s t-test (SPSS program; version 12.0). The average and standard deviations (SDs) of the data are presented. A significance level of *p* < 0.05 was used to determine whether the observed differences were statistically significant.

Data from in vivo experiments were presented as values ± standard deviations (mean ± SD). GraphPad Prism 5.0 Demo (GraphPad Software, San Diego, CA, USA) was used to perform the statistical analysis of multiple groups. The data groups were compared using one-way analysis of variance (ANOVA) and Tukey’s multiple comparison test. A significance level of *p* < 0.05 indicated statistical significance for observed differences.

## 5. Conclusions

In conclusion, button technology was examined through the use of the solvent change method with the aid of Tween 80 as a stabilizer. No expensive polymers or complicated techniques were required. All in vitro characterization tests emphasized the successful transformation of ORL into nanosized particles and enhanced aqueous solubility. The radiochemical labeling of ORL/ORL-NC using Technetium-99m was optimized as an analytical tool to investigate the pharmacokinetic and biodistribution of the preparations in mice effectively by counting the gamma radiation and calculating the % dose/gram tissue/fluid. The oral absorption and tissue biodistribution of ORL-NC were enhanced compared with ORL, with special tissue distribution to the liver, which can be explained as passive tissue targeting. Enhanced absorption of ORL-NC was approved by the significant reduction in the % of drug concentration/gram tissue of the stomach. The results show a successful technique to develop both the solubility and bioavailability of a challenging low-solubility drug and a promising anticancer drug candidate. Furthermore, the results can be used for further investigation of the beneficial effect of ORL on different organs.

## Figures and Tables

**Table 1:** Particle size (nm), zeta potential (mV), and saturated solubility (µg/mL) of ORL, ORL-NC, and ORL-BC *.

				S.S (Mean ± SD, *n* = 3, µg/mL)	
	Particle Size (nm)	Zeta Potential (mV)	PDI	Water	0.1 N HCl
ORL−NC	329.59 ± 120.75	−27.04	0.277	363.5 ± 43.37	457.57 ± 90.35
ORL−BC	557.63 ± 189.63	−26.19	0.310	60.2 ± 11.24	36.77 ± 5.29

* ORL: orlistat, ORL-NC: orlistat nanocrystals, and ORL-BC: blank crystals.

**Table 2:** DSC of ORL, ORL-NC, and ORL-BC *.

	$T_{m}$ Melting Point °C	Heat Flow (mV)
ORL	45.67	32.204
ORL-NC	47.22	28.595
ORL-BC	49.48	30.62

* ORL: orlistat, ORL-NC: orlistat nanocrystals, and ORL-BC: blank crystals.

**Table 3:** The percentage of drug dissolved and % dissolution efficiency of ORL, ORL-BC, and Orl-NC in 0.1 N HCL at 37 °C ± 0.5 °C *.

	%Q30 (Mean ± SD, *n* = 3)	%Q60 (Mean ± SD, *n* = 3)	%DE 30 min	%DE 60 min
ORL	00.0	00.0	-	-
ORL-NC	70.67 ± 10.89	99.68.0 ± 19.43	39.70	66.18
ORL-BC	46.21 ± 4.59	56.88 ± 4.59	23.93	37.95

* ORL: orlistat, ORL-NC: orlistat nanocrystals, and ORL-BC: blank crystals.

**Table 4:** Optimum conditions for radiolabeling of ORL and ORL-NC *.

Factor	Optimum for Plain ^99m^Tc-ORL (98.1%)	Optimum for Nano-^99m^Tc-ORL-NC (84.8%)
Substrate (ORL or ORL-NC)	3 mg plain ORL	1 mg
Reducing agent Na_2_S_2_O_4_	2 mg	5 mg
Reaction time	90 min	60 min
pH of the reaction medium	7	7

* ORL: orlistat, ORL-NC: orlistat nanocrystals, and ORL-BC: blank crystals.

**Table 5:** Biodistribution of oral ^99m^Tc-ORL solution in normal mice (*n* = 3, mean ± SD) *.

Organs and Body Fluids	% Orally Administered Dose/Gram Tissue at Different Time Intervals			
	1/2 h	1 h	2 h	4 h
Blood	0.60 ± 0.03	2.30 ± 0.30 *	2.70 ± 0.03 *	2.10 ± 0.30 *
Bone	0.40 ± 0.20	1.60 ± 0.20 *	1.90 ± 0.01 *	1.50 ± 0.10 *
Muscle	0.40 ± 0.04	1.40 ± 0.10	1.60 ± 0.20 *	1.50 ± 0.10
Liver	0.30 ± 0.05	0.80 ± 0.07 *	0.90 ± 0.02	1.20 ± 0.20
Stomach	42.0 ± 3.40	33.50 ± 2.60 *	33.30 ±2.20 *	22.3 ± 1.60 *
Intestine	6.20 ± 0.50	16.50 ± 1.10 *	21.60 ± 1.40 *	35.0 ± 2.10 *
Lung	1.30 ± 0.12	4.70 ± 0.20 *	1.90 ± 0.01 *	1.10 ± 0.02 *
Heart	3.00 ± 0.30	3.50 ± 0.30 *	2.10 ± 0.20 *	1.30 ± 0.04 *
Spleen	2.50 ± 0.20	3.20 ± 0.40	2.80 ± 0.05 *	2.40 ± 0.40
Kidney	1.00 ± 0.06	3.00 ± 0.20 *	4.50 ± 0.20 *	3.20 ± 0.30 *
Urine	3.40 ± 0.20	7.50 ± 0.30 *	10.30 ± 1.00 *	14.10 ± 1.30 *
Brain	3.00 ± 0.60	4.10 ± 0.40 *	3.90 ± 0.02 *	1.70 ± 0.03 *
Thyroid	3.00 ± 0.20	3.10 ± 0.02	2.20 ± 0.05 *	1.50 ± 0.05

* Significant difference at *p* < 0.05. ORL: orlistat.

**Table 6:** Biodistribution of oral ^99m^Tc-ORL-NC in normal mice (*n* = 3, mean ± SD) *.

Organs and Body Fluids	%Dose/g Tissue at Different Time Intervals			
	1/2 h	1 h	2 h	4 h
Blood	4.00 ± 0.30	5.60 ± 0.30 *	6.30 ± 0.30 *	5.50 ± 0.30 *
Bone	1.10 ± 0.20	1.50 ± 0.20 *	1.60 ± 0.01	1.50 ± 0.01
Muscle	1.60 ± 0.04	1.80 ± 0.10	3.30 ± 0.20 *	2.20 ± 0.10
Liver	1.20 ± 0.05	2.90± 0.20 *	3.30 ± 0.20	3.50 ± 0.02
Stomach	16.00 ± 1.40	25.0 ± 1.60 *	20.3 ±1.20 *	12.5 ± 1.60 *
Intestine	12.0 ± 0.30	20.0 ± 0.10 *	26.60 ± 0.4 *	18.0 ± 0.10 *
Lung	3.90 ± 0.12	3.50 ± 0.20 *	2.10 ± 0.01 *	2.10 ± 0.20 *
Heart	5.60± 0.30	4.10± 0.30 *	3.30 ± 0.20 *	3.00 ± 0.40 *
Spleen	0.80 ± 0.10	2.0 ± 0.1	3.10 ± 0.30	4.1 ± 0.3
Kidney	1.4 ± 0.6 *	3.0 ± 0.3 *	4.1 ± 0.2 *	4.2 ± 0.3 *
Urine	3.4 ± 0.6 *	7.5 ± 0.3 *	11.3 ± 1.0	14.1 ± 1.3 *
Brain	3.6 ± 0.6 *	5.0 ± 0.3 *	3.0 ± 0.2 *	2.2 ± 0.2 *
Thyroid	2.7 ± 0.01 *	4.0 ± 0.02	3.3 ± 0.05 *	2.5 ± 0.02

* Significant difference at *p* < 0.05. ORL-NC: orlistat nanocrystals.

**Table 7:** Pharmacokinetic parameters of ORL in mice (*n* = 3, mean ± SD) * in different body tissues following the delivery of single oral dosages (30 mg/kg) of ^99m^Tc-ORL and ^99m^Tc-ORL-NC.

Organ	^99m^Tc-ORL			^99m^Tc-ORL-NC		
	*C*_max_ (% Dose/g Tissue)	$T_{max}$ (h)	$AUC_{0\to 4}$ (% Dose/g TISSUE * h)	*C*_max_ (% Dose/g Tissue)	$T_{max}$ (h)	$AUC_{0\to 4}$ (% Dose/g Tissue * h)
Blood	2.7	2	8.175	6.3 *	2	21.15 *
Bone	1.9	2	5.75	1.6 *	2	5.575 *
Muscle	1.6	2	5.15	3.3 *	2	9.3 *
Liver	1.2	4	3.3	3.5 *	4	11.225 *
Stomach	42	0.5	118.375	25 *	2	69.7 *
Intestine	35	4	82.875	26.6 *	2	78.9 *
Lung	4.7	1	8.125	3.9 *	0.5	9.825 *
Heart	3.5	1	8.575	5.6 *	0.5	13.825 *
Spleen	3.2	1	10.25	4.1 *	4	10.65 *
Kidney	4.5	2	12.7	4.2 *	4	13.3 *
Urine	14.1	4	36.875	14.1	4	38.375 *
Brain	4.1	1	12.125	5 *	1	17.47 *
Thyroid	3.1	1	8.625	4 *	1	11.8 *

* Significant difference at *p* <  0.05. ORL: orlistat and ORL-NC: orlistat nanocrystals.

**Table 8:** Effect of ORL and ORL-NC on serum liver function biomarkers.

Group	SGPT (U/L)	SGOT (U/L)	ALP (U/L)	Total Bilirubin (mg/dL)	Direct Bilirubin (mg/dL)
Normal control	41.83 ± 2.31	40.17 ± 2.92	119.2 ± 4.021	0.68 ± 0.11	0.36 ± 0.052
SEC control	129 ± 5.06 *	130 ± 4.85 *	408.2 ± 7.44 *	1.68 ± 0.15 *	1.8 ± 0.14 *
SEC+TAM	70.67 ± 3.55 *^,a^	71.5 ± 7.17 *^,a^	183.2 ± 9.51 *^,a^	0.96 ± 0.052 *^,a^	0.9 ± 0.059 *^,a^
SEC+ORL	102.3 ± 2.16 *^,a,b^	109 ± 3.74 *^,a,b^	275.8 ± 5.11 *^,a,b^	1.34 ± 0.083 *^,a,b^	1.3 ± 0.059 *^,a,b^
SEC+ORL-NC	81.17 ± 4.62 *^,a,c^	85.33 ± 5.78 *^,a,c^	202.8 ± 6.01 *^,a,c^	1.13 ± 0.095 *^,a,c^	1.1 ± 0.072 *^,a,c^

SEC mice treated with the control vehicles, TAM (tamoxifen 10 mg/kg, IP), ORL (orlistat 240 mg/kg, IP), and ORL-NC (orlistat nanocrystals 240 mg/kg, IP). All drugs or their vehicles were administered daily for 14 days starting from day 12 after tumor implantation. Data are presented as mean ± SD, *n* = 6. * Significant difference from the normal control group at *p* < 0.05. ^a^ Significant difference from the SEC control group at *p* < 0.05. ^b^ Significant difference from SEC+TAM group at *p* < 0.05. ^c^ Significant difference from SEC+ORL group at *p* < 0.05.

## Data Availability

Data is contained within the article.
